# The efficacy of adjunctive *Garcinia mangostana* Linn. (mangosteen) pericarp extract for bipolar depression: 24-week randomised controlled trial

**DOI:** 10.1192/bjp.2025.108

**Published:** 2025-12

**Authors:** Olivia M. Dean, Susan M. Cotton, Melanie M. Ashton, Alyna Turner, Lucy Saunders, Chee H. Ng, Malcolm Hopwood, Seetal Dodd, Jon-Paul Khoo, Adam J. Walker, Mary Lou Chatterton, Bianca E. Kavanagh, Sarah E. Nadjidai, Samantha L. Lo Monaco, Brian H. Harvey, Gin S. Malhi, Ellie Brown, David R. Skvarc, Danica Diao, Felice N. Jacka, Jerome Sarris, Nathan L. Dowling, Michael Berk

**Affiliations:** IMPACT Strategic Research Centre, School of Medicine, Deakin University, Barwon Health, Geelong, Australia; Florey Institute for Neuroscience and Mental Health, University of Melbourne, Parkville, Australia; Orygen Ltd, The National Centre of Excellence in Youth Mental Health, Parkville, Australia; Centre of Youth Mental Health, The University of Melbourne, Parkville, Australia; School of Psychological Sciences, Monash University, Clayton, Australia; Turner Institute for Brain and Mind, Clayton, Australia; Department of Psychiatry University of Melbourne, The Melbourne Clinic, Professorial Unit, Richmond, Australia; Department of Psychiatry University of Melbourne, Albert Road Clinic, Melbourne, Australia; Faculty of Medicine, The University of Queensland, Brisbane, Australia; Deakin University, Institute for Health Transformation, Geelong, Australia; Deakin Rural Health, School of Medicine, Deakin University, Warrnambool, Australia; Center of Excellence for Pharmaceutical Sciences, School of Pharmacy, North-West University, Potchefstroom, South Africa; MRC Unit on Risk and Resilience in Mental Disorders, Department of Psychiatry and Neuroscience Institute, University of Cape Town, Cape Town, South Africa; Discipline of Psychiatry, Sydney Medical School, University of Sydney, Sydney, Australia; CADE Clinic, Department of Psychiatry, Royal North Shore Hospital, St. Leonards, Australia; Department of Psychiatry, University of Oxford, Oxford, UK; NICM Health Research Institute, Western Sydney University, Westmead, Australia; Centre for Mental Health, Swinburne University, Melbourne, Australia

**Keywords:** *Garcinia mangostana*, mangosteen pericarp extract, bipolar disorder, clinical trial, depression

## Abstract

**Background:**

Bipolar depression remains difficult to treat, and people often experience ongoing residual symptoms, decreased functioning and impaired quality of life. Adjunctive therapies targeting novel pathways can provide wider treatment options and improve clinical outcomes. *Garcinia mangostana* Linn. (mangosteen) pericarp has serotonogenic, antioxidant anti-inflammatory and neurogenic properties of relevance to the mechanisms of bipolar depression.

**Aims:**

The current 28-week randomised, multisite, double-blind, placebo-controlled trial investigated mangosteen pericarp extract as an adjunct to treatment-as-usual for treatment of bipolar depression.

**Method:**

This trial was prospectively registered on the Australia New Zealand Clinical Trials Registry (no. ACTRN12616000028404). Participants aged 18 years and older with a diagnosis of bipolar I or II and with at least moderate depressive symptoms were eligible for the study. A total of 1016 participants were initially approached or volunteered for the study, of whom 712 did not progress to screening, with an additional 152 screened out. Seventy participants were randomly allocated to mangosteen and 82 to a placebo control. Fifty participants in the mangosteen and 64 participants in the placebo condition completed the treatment period and were analysed.

**Results:**

Results indicated limited support for the primary hypothesis of superior depression symptom reduction following 24 weeks of treatment. Although overall changes in depressive symptoms did not substantially differ between conditions over the course of the trial, we observed significantly greater improvements for the mangosteen condition at 24 weeks, compared with baseline, for mood symptoms, clinical impressions of bipolar severity and social functioning compared with controls. These differences were attenuated at week 28 post-discontinuation assessment.

**Conclusions:**

Adjunctive mangosteen pericarp treatment appeared to have limited efficacy in mood and functional symptoms associated with bipolar disorder, but not with manic symptoms or quality of life, suggesting a novel therapeutic approach that should be verified by replication.

Bipolar disorder is a complex illness that usually requires a multimodal treatment approach encompassing medication (mood stabilisers, antidepressants, antipsychotics), psychotherapy, brain stimulation and lifestyle modification to achieve optimal outcomes.^1^ However, despite these evidenced-based treatment options, a significant proportion of individuals experience a discernible shortfall in functional recovery following a mood episode and subsequent treatment. The depressive phase of the illness is particularly challenging, because available treatments are less effective compared with management of mania. This disparity is also accentuated by the fact that people with bipolar disorder are most vulnerable to suicide when depressed.^2^ Therefore, there is an urnt need to identify new treatments for bipolar depression that not only assist in symptom remission but also facilitate functional recovery.

Despite the acknowledged role of glutamate, dopamine, serotonin and noradrenaline in the biology of bipolar disorder and their role in the assumed mode of action of current agents, the lack of optimal efficacy of these said agents emphasises the fact that other processes are involved.^3^ Indeed, agents with multimodal actions on neurotransmitter and redox inflammatory systems would seem to offer distinct benefits for the long-term treatment outcome of mood and psychotic disorders. In this regard, mitochondrial dysregulation and its bidirectional impact on inflammatory and redox pathways^3,4^ is of particular significance. It is not unusual that a variety of plant and herbal preparations offer multimodal actions.^5^ The serotonergic, antioxidant, anti-inflammatory and neurogenic properties of mangosteen pericarp^6^ are therefore considerably relevant to the biological mechanisms underpinning bipolar depression. Mangosteen pericarp contains several bioactive constituents, including alpha-mangostin, gamma-mangostin and gartanin. Importantly, the 5-HT2A receptor-blocking actions of some constituents of mangosteen pericarp^6^ not only offer potential efficacy for depressed mood and negative symptom schizophrenia, e.g. clozapine,^3^ but also selective targeting of these receptors may also produce clinically relevant antidepressant effects (e.g. mirtazapine).^3^

## The current study

The current randomised controlled trial investigates an extract of *Garcinia mangostana* Linn. (mangosteen) pericarp as a potential adjunctive novel therapy for the treatment of bipolar depression.^6^ The rationale for exploring mangosteen’s potential as a treatment for bipolar depression is supported by the current understanding of the underlying pathophysiology of the disorder, and by preclinical studies directly testing mangosteen and its bioactive compounds.^6,7^

In the mood–psychosis continuum, inflammation has emerged as a central role player in the pathophysiology of major depression, schizophrenia and bipolar disorder, with levels of pro-inflammatory cytokines increased in these conditions, as well as a decrease in anti-inflammatory cytokines, contributing to the inflammatory component of these disorders (see Brand et al for review).^3^ Preclinical evidence suggests that chronic mangosteen pericarp (50 mg/kg/day) displays significant antidepressant and pro-cognitive effects in a genetic rodent model of depression, demonstrating parity with imipramine.^8^ In line with its monoaminergic profile, behavioural and regional brain monoamine assessments suggest a more prominent serotonergic action for mangosteen extract.^8^ That said, both depressive and psychosis-like manifestations induced in a prenatal maternal inflammation model in rats showed that depressive-like behaviour responded favourably to raw mangosteen extract (as well as to alpha-mangostin, and combinations with haloperidol), while psychosis-like manifestations did not.^9^ The latter findings suggest preferential antidepressant- over antipsychotic-like actions. Nevertheless, the former effects occurred simultaneously with reversal of lipid peroxidation in limbic brain regions, and a lowering of elevated plasma pro-inflammatory cytokines,^8,9^ congruent with anti-inflammatory actions. Taken together, these animal studies provide proof of concept for testing the efficacy of mangosteen extract in bipolar depression.

## Primary hypothesis

That 24 weeks of adjunctive mangosteen pericarp treatment would be superior to placebo in reducing depression symptoms, between baseline and week 24, measured using the Montgomery–Asberg Depression Rating Scale (MADRS).

## Secondary hypotheses

(a) That 24 weeks of adjunctive mangosteen pericarp treatment would be superior to placebo in reducing the symptom domains of bipolar illness measured, including mood symptoms, mania, clinical impressions, quality of life and general functioning.

(b) That those receiving mangosteen pericarp treatment would have better outcomes 4 weeks post-treatment discontinuation (week 28), based on change in symptom severity, quality of life and functioning scales, than those taking the placebo.

## Method

A protocol for this randomised controlled trial was published^7^ and the trial was prospectively registered on the Australian and New Zealand Clinical Trials Registry (no. ACTRN12616000028404). Blood samples were collected and cognitive function was measured, although these have yet to be analysed and are not reported here. Similarly, personality traits, diet behaviours and other tertiary data were collected but are not reported here.

### Investigational product

Participants took 2 × 500 mg of mangosteen pericarp extract capsules (VitalXan, Australia) once a day for 24 weeks. Placebo and mangosteen pericarp capsules were produced simultaneously by the same manufacturer according to Good Manufacturing Practice (PCI Pharma Services, Australia) and were matched in size and appearance. Bottles were dispensed every 4 weeks, and any unused medication was returned by participants.

### Study design

Participants were recruited from four trial sites in Australia, located at Geelong and Melbourne (Victoria) and Brisbane (Queensland). The Geelong site recruited from the Barwon Health Mental Health and Drug and Alcohol outpatient service. The two Melbourne sites (The Melbourne Clinic and The Albert Road Clinic) and the Brisbane site (Toowong Specialist Clinic) are private clinics. All sites additionally recruited from the community and via advertising (online, radio and printed media). Each participant attended a screening interview, where written informed consent was obtained and subsequently randomised. Participants were given AU$20 travel reimbursement at each post-randomisation interview.

Recruitment was conducted between 6 June 2016 and 3 November 2020 (disrupted over the COVID-19 pandemic). Face-to-face interviews were also disrupted by the COVID-19 pandemic. Participant interviews were conducted online, and participants continued to receive trial medication and altered safety procedures.

The authors assert that all procedures contributing to this work comply with the ethical standards of the relevant national and institutional committees on human experimentation, and with the Helsinki Declaration of 1975 as revised in 2013. All procedures involving human subjects/patients were approved by the following Human Research and Ethics Committees; Barwon Health (no. 15/124), Deakin University (no. 2015/097) and The Melbourne Clinic (no. 262). The design of the study’s protocol is concordant SPIRIT.^10^

### Randomisation

Allocation to mangosteen or placebo was conducted using a 1:1 permutated block-of-four randomisation. The participants, researchers and statistician were blind to group allocation. An independent researcher generated the randomisation code applied by the manufacturer to the active ingredient or placebo, prior to being received by the trial pharmacy at each site.

### Inclusion

Participants were required to be aged 18 years or over, have the capacity to consent to the study and to follow the instructions and procedures, be fluent in English, have a current treating physician and fulfill the DSM-5^11^ diagnosis of bipolar disorder I or II, or another specified bipolar and related disorder and current major depressive episode, with at least moderate depressive symptoms (MADRS ≥20). Participants’ pre-existing psychotropic therapy regimens were required to have been stable for 4 weeks prior to study entry. Bipolar disorder and other psychiatric comorbidities were assessed using the Structured Clinical Interview for DSM-5.^12^ MADRS was readministered if there was a delay of >7 days between screening and baseline interviews, or between baseline interview and medication commencement. Potential participants with known or suspected clinically unstable systemic medical disorder; contraindications or intolerance to mangosteen pericarp, pregnant or breastfeeding; or current enrollment in another clinical trial were excluded.

### Withdrawal

Participants were withdrawn from the trial if they removed consent, became pregnant, commenced new electroconvulsive therapy treatment, ceased trial medication for 7 consecutive days or reported an increase in suicidal ideation. Withdrawal due to suicidal ideation or adverse events was at the discretion of the research team or the participant, or at the request of the treating physician. Changes to existing treatment regimens were recorded.

### Efficacy measures

The published protocol includes a table of the full procedures and validated rating scales administered at each trial interview.^7^

MADRS^13^ (the primary outcome measure), Bipolar Depression Rating Scale^14^ (BDRS), Hamilton Anxiety Scale^15^ (HAM-A), Young Mania Rating Scale^16^ (YMRS), Clinical Global Impression Scale for Bipolar Disorder-Scale^17^ (CGI-BP) and Patient Global Impression Scale^18^ (PGI) were used to assess overall bipolar disorder symptoms.

Functioning and quality of life was assessed by the Social and Occupational Functioning Scale^19^ (SOFAS), the Range of Impaired Functioning Tool^20^ (LIFE-RIFT) and Quality of life Enjoyment and Satisfaction Questionnaire – Short Form^21^ (Q-LES-Q-SF).

At every visit, participants were asked open-ended questions about their trial experience and all adverse events were reviewed by a principal investigator. The study Data Safety Monitoring Board reviewed all adverse events. The research team followed up ongoing adverse events recorded at the final week 28 visit to determine the outcome of the event. Serious adverse events were reported according to Good Clinical Practice guidelines. The clinical trial data were collected and managed using REDCap.^22^

### Statistical analysis

We used a mixed-model, repeated-measures approach with average treatment group differences for the primary outcome measure up to week 28. Within this model the fixed variables were treatment group, site, visit and treatment-by-visit interaction. Primary efficacy was analysed using planned comparisons of between-group differences in depressive symptomatology from baseline to week 24. To determine post-discontinuation effects, the difference between weeks 24 and 28 was tested using planned comparisons. We performed *a priori* power calculations to determine a target sample size of 150 participants. For a two-tailed analysis with *α* = 0.05, *Zα* = 1.96 and with *β* = 0.2, *Zβ* = 0.8 and *n* = 120, the trial was powered at 80% to detect a true difference in MADRS scores between the mangosteen pericarp and placebo groups (assuming Cohen’s *d* = 0.362 or greater). This is a conservative estimate, based on the results of pilot data^23^ and our previous trial of *N*-acetylcysteine specifically investigating bipolar depression,^24^ in which effect sizes for depressive symptoms (BDRS, MADRS) were similar to or above the proposed trial. An attrition rate of 20% was estimated for participants with no post-baseline data,^25^ and therefore 150 participants were required.

## Results

Table 1 lists the demographic and clinical characteristics of the total cohort of 152 participants (mangosteen, *n* = 70; placebo, *n* = 82) who were randomised. Most of the cohort were female (65%) and were aged between 18 and 77 years of age (*M* = 42.8, s.d. = 13.2). There was a delay of approximately 10 years between onset of symptoms and diagnosis. The number of past depressive episodes in the cohort ranged from 0 to 150, with an average of 30.9 (s.d. = 30.2).

**Table 1:** Baseline demographic and clinical characteristics of the total cohort and separately for the two treatment groups

Variable	Total	Mangosteen	Placebo
	(*N* = 152)	(*n* = 70)	(*n* = 82)
Female gender % (*n*)	65.8 (100)	67.1 (47)	64.6 (53)
Age *M* (s.d.)	42.8 (13.2)	41.6 (13.7)	43.8 (12.8)
Married or de facto % (*n*)	45.0 (68)	41.4 (29)	48.1 (39)
Australian born % (*n*)	84.9 (129)	85.7 (60)	84.1 (69)
Highest education level			
Years 10–11 % (*n*)	8.0 (12)	8.7 (6)	7.4 (6)
Year 12 % (*n*)	19.3 (29)	18.8 (13)	19.8 (16)
Bachelor’s degree % (*n*)	31.3 (47)	33.3 (23)	29.6 (24)
Postgraduate degree % (*n*)	15.3 (23)	13.0 (9)	17.3 (14)
Certificate, diploma, apprenticeship or other TAFE % (*n*)	26.0 (39)	26.1 (18)	25.9 (21)
Psychiatric history			
Age of first symptoms *M* (s.d.)	18.5 (9.5)	17.5 (8.9)	19.4 (9.9)
Age of diagnosis *M* (s.d.)	33.3 (11.5)	32.5 (11.6)	34.0 (11.4)
Duration of illness (self-report) *M* (s.d.)	24.0 (12.5)	24.2 (13.2)	23.9 (12.0)
Duration of illness (since diagnosis) *M* (s.d.)	9.6 (8.3)	9.0 (8.8)	10.0 (7.9)
Number of depressive episodes *M* (s.d.)	30.9 (30.2)	34.0 (31.4)	28.2 (29.1)
Number of psychiatric hospitalisations *M* (s.d.)	2.7 (4.8)	2.8 (4.8)	2.6 (4.9)
Number of suicide attempts			
0 % (*n*)	55.3 (84)	57.1 (40)	53.7 (44)
1 % (*n*)	17.8 (27)	17.1 (12)	18.3 (15)
2 or more % (*n*)	27.0 (41)	25.7 (18)	28.0 (23)
Substance use			
Cigarette use (past month) % (*n*)	21.7 (33)	21.4 (15)	22.0 (18)
Alcohol use (past month) % (*n*)	65.8 (100)	68.6 (48)	63.4 (52)
Drugs use (past month) % (*n*)	17.1 (26)	18.6 (13)	15.9 (13)

TAFE, Technical and Further Education.

### Participant flow

**Fig. 1:**
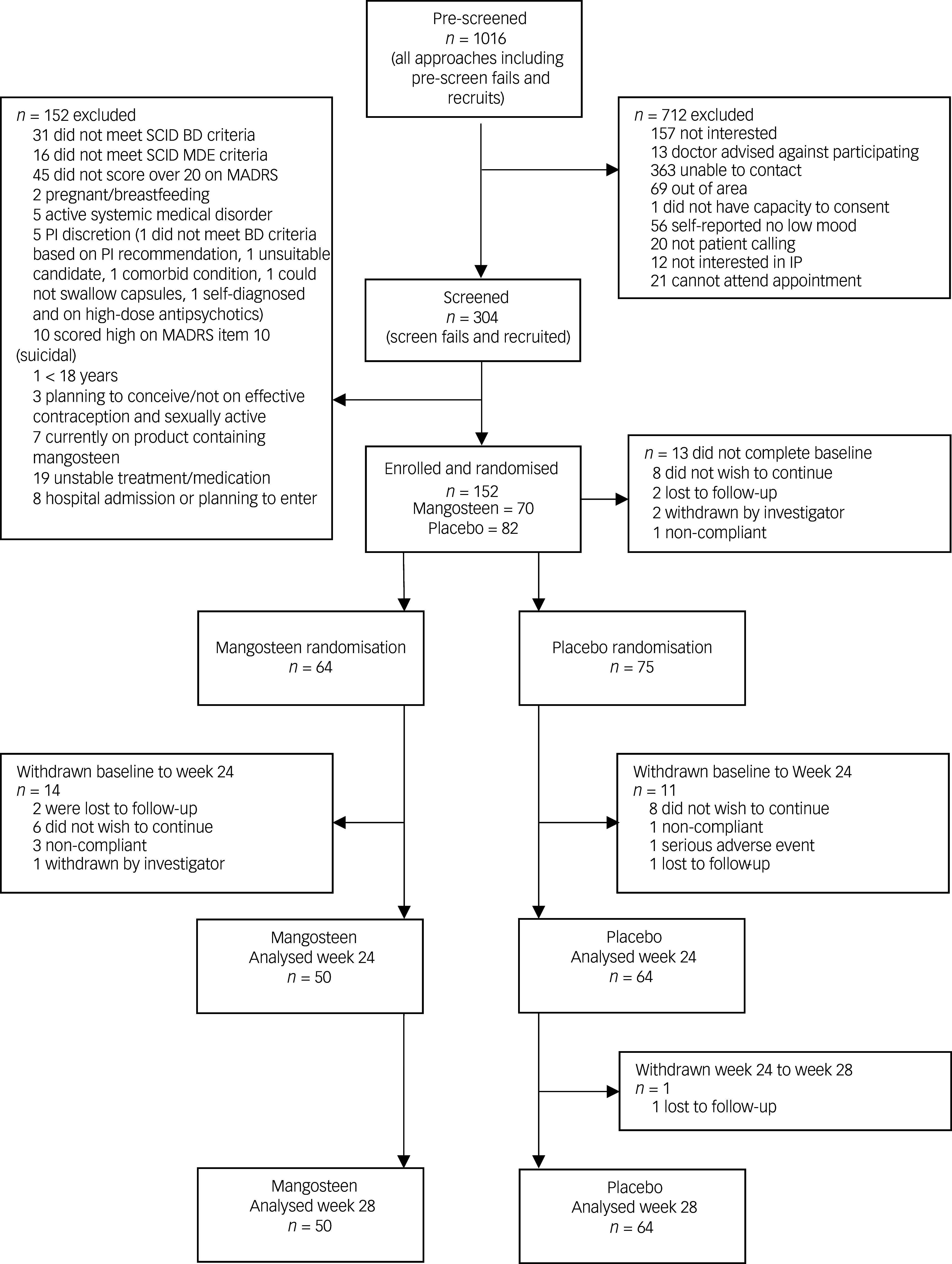
CONSORT diagram outlining participant flow from identification and screening through to enrollment and withdrawals. Overall, from baseline to the end of the treatment phase (week 24, the primary endpoint), 75% of participants completed the trial. SCID, Structured Clinical Interview for DSM-IV; MADRS, Montgomery–Asberg Depression Rating Scale; BD, bipolar disorder; IP, investigational product; MDE, major depressive episode; PI, principal investigator.

Participant flow can be found in the CONSORT^26^ flowchart in Fig. 1. There were 14 individuals who withdrew after baseline, and 25 who had post-baseline data but did not complete the study.

Non-completers were significantly younger at onset of symptoms (*M* = 14.7, s.d. = 5.2) than completers (*M* = 19.0, s.d. = 10.0), *t*(69.74) = 3.05, *P* = 0.003. Non-completers were also significantly more likely to have used tobacco (non-completers, 36.0% [*n* = 9]; completers, 17.7% [*n* = 20], *χ* ^2^ [1] = 4.13, *P* = 0.042) and alcohol (non-completers, 84.0% [*n* = 21]; completers, 60.2% [*n* = 69], *χ* ^2^ [1] = 5.07, *P* = 0.024) in the month prior to baseline. There were no significant differences between these groups on any of the symptom measures.

### Trial medication adherence

The overall adherence to trial medication was high, with a median of 80.3% (interquartile range [IQR] = 64.6–85.2%). Capsule audit revealed a median discrepancy between expected and observed returned capsules of 8 (IQR = 7–9) capsules at each visit, indicating that most participants had missed around 4 days’ worth of medication between each visit.

### Inter-rater reliability

Inter-rater reliability was checked across seven independent raters for each scale. Inter-rater reliability was found to be good to excellent across each of the outcome measures: *κ* = 0.92, 0.9, 0.91, 0.95, 0.96 and 0.78 for MADRS, BDRS, HAM-A, YMRS, SAPAS and LIFE-RIFT, respectively.

### Primary efficacy outcome – depressive symptoms

**Fig. 2:**
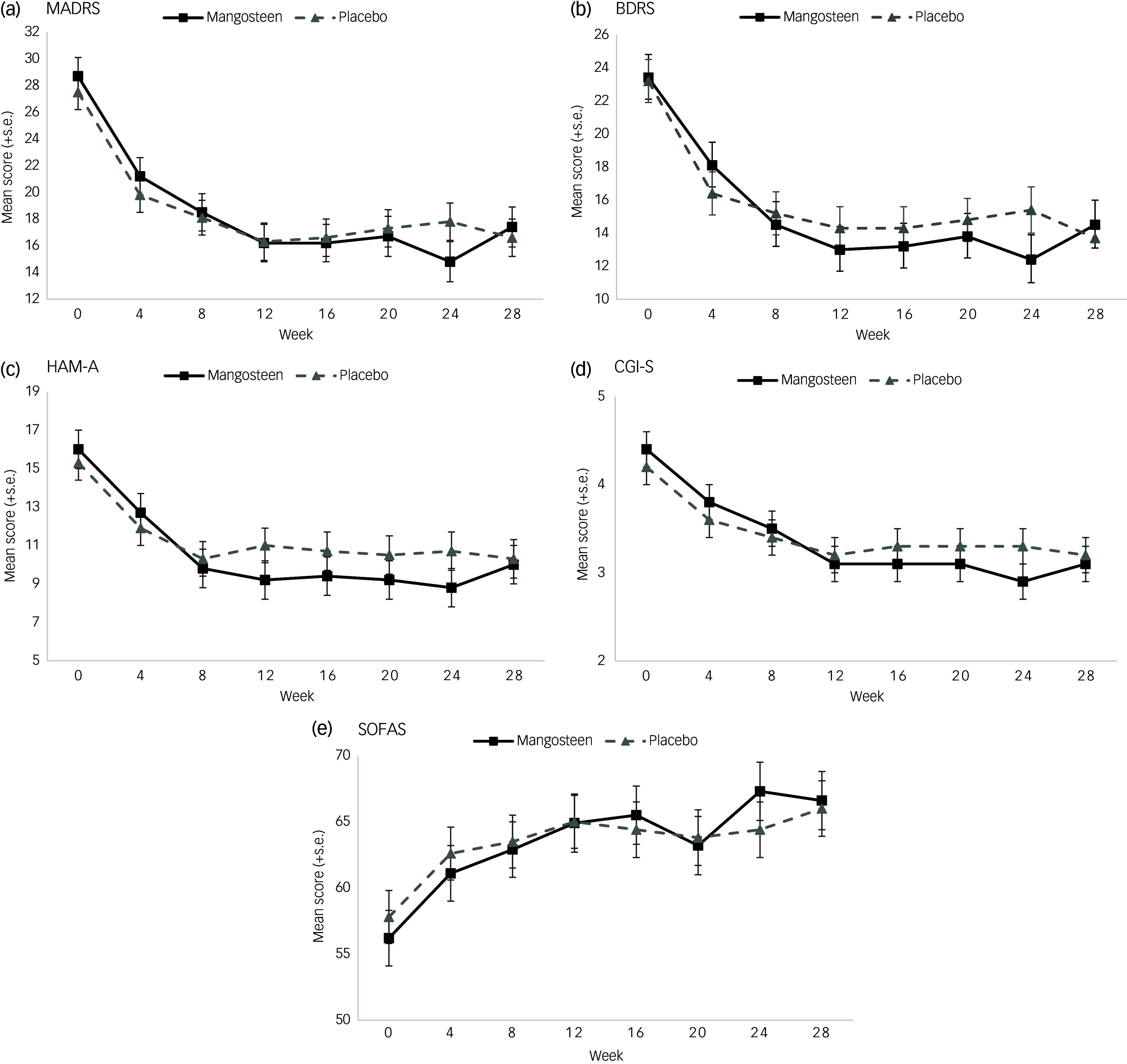
Between-group differences (*M* ± s.e.) as estimated from mixed-model repeated measures on (a) Montgomery–Asberg Depression Rating Scale (MADRS); (b) Bipolar Depression Rating Scale (BDRS); (c) Hamilton Anxiety Scale (HAM-A); (d) Clinical Global Impression Scale – Severity (CGI-S); and (e) Social and Occupational Functioning Scale (SOFAS). Hospital Anxiety and Depression Scale from baseline to week 28.

Figure 2(a) demonstrates the differences between the two groups on the MADRS scale over the 28 weeks of the study, including 24 weeks of treatment and the 4-week post-discontinuation period. The groups demonstrated similar rates of change in depressive symptoms until week 20 treatment. As such, the interaction between group and visit was not significant: *F*(7, 318.8) = 1.02, *P* = 0.414, but the visit main effect was significant: *F*(7, 319.0) = 34.30, *P* < 0.001, with symptoms declining over time. Notable in Fig. 2(a) is that the trajectory of depressive symptoms between the two groups diverged between 20 and 24 weeks. We observed that the rate of change from baseline to the primary endpoint (24 weeks) on the MADRS was significantly greater in the mangosteen (*M* = 13.8, s.e. = 1.5) than in the placebo group (*M* = 9.7, s.e. = 1.3), *t*(228.3) = 2.10, *P* = 0.037 (see Table 2).

**Table 2:** Group differences on primary and secondary outcomes at baseline and at the primary endpoint (24 weeks)

							Primary efficacy		Post-discontinuation	
Outcome measure	Mangosteen			Placebo			Group differences in rate of change from baseline to week 24	*P*-value	Group differences in rate of change from week 24 to week 28	*P*-value
	Baseline	Week 24	Week 28	Baseline	Week 24	Week 28	*M*_diff_ (s.e._diff_)		*M*_diff_ (s.e._diff_)	
Primary outcome										
MADRS	28.7 (1.4)	14.8 (1.5)	17.4 (1.5)	27.5 (1.3)	17.8 (1.4)	16.6 (1.4)	4.2 (2.0)	0.037	−3.7 (1.8)	0.037
Secondary outcomes										
Symptoms										
BDRS	23.4 (1.4)	12.4 (1.5)	14.5 (1.5)	23.2 (1.3)	15.4 (1.4)	13.7 (1.4)	3.1 (1.9)	0.096	−3.7 (1.7)	0.028
YMRS	2.5 (0.5)	2.6 (0.6)	3.0 (0.6)	2.9 (0.5)	3.0 (0.5)	3.0 (0.6)	−0.1 (0.8)	0.902	−0.4 (0.8)	0.634
HAM-A	16.0 (1.0)	8.8 (1.0)	10.0 (1.0)	15.3 (0.9)	10.7 (1.0)	10.3 (1.0)	2.5 (1.2)	0.039	−1.6 (1.1)	0.137
CGI-S	4.4 (0.2)	2.9 (0.2)	3.1 (0.2)	4.2 (0.2)	3.3 (0.2)	3.2 (0.2)	0.6 (0.3)	0.029	−0.3 (0.2)	0.220
Functioning										
SOFAS	56.2 (2.1)	67.3 (2.2)	66.6 (2.2)	57.8 (2.0)	64.4 (2.1)	65.9 (2.1)	−4.5 (2.2)	0.040	2.2 (1.8)	0.203
LIFE-RIFT	12.7 (0.5)	10.1 (0.5)	10.7 (0.5)	13.0 (0.5)	10.8 (0.5)	10.7 (0.5)	0.3 (0.6)	0.594	−0.7 (0.5)	0.161
Quality of life										
Q-LES-Q-SF	45.5 (2.5)	61.6 (2.6)	58.4 (2.6)	44.9 (2.3)	59.2 (2.4)	59.1 (2.5)	−1.8 (3.1)	0.574	3.0 (2.7)	0.265

Data are means, with standard errors in parentheses. MADRS, Montgomery–Åsberg Depression Rating Scale; BDRS, Bipolar Depression Rating Scale; YMRS, Young Mania Rating Scale; HAM-A, Hamilton Anxiety Rating Scale, CGI-S, Clinical Global Impression Scale – Severity; SOFAS, Social and Occupational Functioning; LIFE-RIFT, Range of Impaired Functioning Tool; Q-LES-Q-SF, Quality of Life Employment and Satisfaction Questionnaire – Short Form.

### Secondary outcomes

There were no significant group-by-visit interactions for all secondary outcome variables, with both groups generally demonstrating similar changes over time, especially up to the week 20 visit. The main effect estimates for visit were significant at *P* < 0.001 for all variables, apart from YMRS (*P* = 0.152).

Three variables were similar to MADRS regarding between-group differences in the rate of change from baseline to week 24. The mangosteen group showed a significantly greater drop in symptoms compared with the placebo group in terms of HAM-A (see Fig. 2(c); mangosteen, *M* = 7.2, s.e. = 0.9; placebo, *M* = 4.7, s.e. = 0.8; *t[*271.99] = 2.08, *P* = 0.039) and Clinical Global Impression Scale – Severity (CGI-S) (see Fig. 2(d); mangosteen, *M* = 1.4, s.e. = 0.2; placebo group, *M* = 0.8, s.e. = 0.2; *t*([216.0] = 2.20, *P* = 0.029). With regard to SOFAS, the mangosteen group (*M* = 11.1, s.e. = 1.6) demonstrated almost double the increase in scores as compared with the placebo group (*M* = 6.6, s.e. = 1.5) from baseline to week 24, with this difference being significant, *t*(216.5) = −2.06, *P* = 0.040 (see Fig. 2(e)).

At week 24, CGI-I was slightly higher in the placebo group (*M* = 2.8, s.e. = 0.1) than in the mangosteen group (*M* = 2.4, s.e. = 0.2); however, this difference was not significant, *t*(106) = −1.97, *P* = 0.051. A similar result was found for PGI-I (mangosteen, *M* = 2.7, s.e. = 0.2; placebo, *M* = 3.1, s.e. = 0.1), *t*(110) = −1.69, *P* = 0.094.

### Post-discontinuation

Descriptive statistics pertaining to change from week 24 to week 28 or the post-discontinuation period can be seen in Table 2 and Fig. 2. For MADRS, the mangosteen group showed a worsening of symptoms (*M* = 2.6, s.e. = 1.3) whereas the placebo group saw a slight improvement (*M* = −1.2, s.d. = 1.2); the difference between the groups in change from week 24 to week 28 was significant, *t*(640.98) = −2.10, *P* = 0.037. Similar trends were noted for BDRS; the mangosteen group showed a worsening of symptoms (*M* = 2.0, s.e. = 1.2) whereas the placebo group showed improvement (*M* = −1.6, s.e. = 1.1), *t*(640.5) = −2.20, *P* = 0.028. No other between-group differences were observed with respect to the post-discontinuation period.

### Adverse events

Overall, 186 individual types of adverse event were reported according to Medical Dictionary for Regulatory Activities (MedDRA) codes. A total of 187 individual adverse events were reported in the mangosteen pericarp group and 217 in the placebo group. Table 3 displays the frequency of adverse events occurring in the mangosteen and placebo groups, according to MedDRA System Organ Class (SOC). The frequency of each symptom is provided in Supplementary Table 1.

**Table 3:** Frequency of each adverse event in the mangosteen and placebo groups

Adverse event organ code	Adverse event symptoms	Placebo	Mangosteen	Total
Ear and labyrinth disorders (10013993)	Tinnitus, vertigo	2	3	5
Endocrine disorders (10014698)	Hypothyroidism	2	1	3
Gastrointestinal disorders (10017947)	Abdominal discomfort, abdominal pain, bloating, blood in stool, constipation, menstrual pain, diarrhoea, diverticulitis, gastroenteritis, gastrointestinal pain, heartburn, indigestion, nausea, stomach ache, vomiting, gastrointestinal upset	34	36	70
General disorders and administration site conditions (10018065)	Breast swelling, fever, sweating, tiredness/fatigue, decreased appetite	12	4	16
Immune system disorders (10021428)	Hay fever	3	2	5
Infections and infestations (10021881)	Infection, sinusitis, urinary tract infection, viral infection, cold sore (mouth)	6	8	14
Investigations (10022891)	Colonoscopy, weight gain, weight loss	6	1	7
Metabolism and nutrition disorders (10027433)	Hyperglycaemia, hypoglycaemia, vitamin D deficiency	8	1	9
Musculoskeletal and connective tissue disorders (10028395)	Back pain, knee pain, muscle pain, fibromyalgia, ligament injury	7	7	14
Nervous system disorders (10029205)	Dizziness, headache, migraine	16	16	32
Psychiatric disorders (10037175)	Depression, depression worsening, elevated mood, hallucination, hypomania, mania, panic attack, psychotic, suicidal ideation	15	19	34
Renal and urinary disorders (10038359)	Dysuria, elevated LFTs	0	4	4
Respiratory, thoracic and mediastinal disorders (10038738)	Chest infection, bloating, cough, upper respiratory infection, cold, cough, flu, sinus infection, sore throat, bronchitis	30	33	63
Skin and subcutaneous tissue disorders (10040785)	Rash	0	2	2
**Total**		**141**	**137**	**278**

LFT, liver function test.

The most commonly reported adverse events were cold (mangosteen, *n* = 16; placebo, *n* = 14), headache (mangosteen, *n* = 7; placebo, *n* = 12), nausea (mangosteen, *n* = 8; placebo, *n* = 9) and diarrhoea (mangosteen, *n* = 9; placebo, *n* = 4). Adverse events with the greatest disparity were tiredness (mangosteen, *n* = 0; placebo, *n* = 5) and vitamin D deficiency (mangosteen, *n* = 5; placebo, *n* = 0). A total of 28 serious adverse events (SAEs) were reported (mangosteen, *n* = 17; placebo, *n* = 11), the most common being suicidal ideation (mangosteen, *n* = 4; placebo, *n* = 3). The number of participants reporting SAEs did not significantly differ between conditions (mangosteen, *n* = 12; placebo, *n* = 9; chi-square *P* = 0.52).

### Concomitant medications

Just over half of all participants reported changes in their regular antidepressants, mood stabilisers or antipsychotics over the period of the trial (*n* = 72, 54.5%). Among placebo participants, *n* = 10/20, 15/32 and 14/20 reported reducing their daily doses of each medication type throughout the trial, compared with *n* = 10/18, 15/23 and 17/25 mangosteen participants (the numerator is the number of participants reporting a reduction, the denominator is the total number of participants reporting a change). There was little evidence of meaningful differences between the treatment conditions (see Supplementary Table 2).

## Discussion

This large multicentre, double-blinded, randomised, placebo-controlled trial investigated the efficacy of adjunctive mangosteen pericarp extract in improving symptoms of bipolar depression. We observed limited support for the primary hypothesis, that 24 weeks of adjunctive mangosteen pericarp treatment would be superior to placebo in reducing depression symptoms, because the mangosteen condition did not significantly differ from placebo for the first 20 weeks. However, the mangosteen condition showed a superior rate of change to placebo at 24 weeks before returning to equilibrium by 28 weeks. Our examination of the secondary outcomes corroborated these findings, whereby we observed no between-condition differences on any outcome at week 28, but superior reduction for the mangosteen condition over the treatment administration period of the trial. Despite the lack of an overall condition-by-visit interaction, according to MADRS, we observed that participants receiving mangosteen pericarp extract experienced a significantly greater reduction in anxiety symptoms at week 24 compared with baseline levels. We also observed comparable effects for clinical improvement and general functioning. Our observations of participants experiencing apparent post-treatment regression of improvements suggest that continued administration of mangosteen is required if any beneficial effects are to be maintained. We also note that mangosteen did not significantly improve the rate of change for all outcomes, and that the superiority of mangosteen for depressive symptoms and psychosocial functioning was inconsistent across measures of those constructs. Notably, although manic symptoms remained consistent across the sample, due to the targeted recruitment of current depression, such symptoms were low in both groups at baseline. In aggregate, mangosteen pericarp extract may have some efficacy in reducing depressive symptoms and improving functioning and quality of life in participants experiencing bipolar depression. These clinical findings are broadly consistent with those observed in a maternal, inflammation-based, animal model of depression and psychosis after the animals received either mangosteen pericarp or alpha mangostin (see Lotter et al^9^).

Considering the multimodel serotonergic, anti-inflammatory and antioxidant actions for mangosteen pericarp described earlier, the significant improvements at week 24 demonstrated in this clinical trial suggest that mangosteen may have a role in the treatment of the depressive phase of bipolar disorder. Our findings are encouraging since, despite many decades of research in mood and psychotic disorders, and the seminal discoveries of drugs including lithium, imipramine and chlorpromazine, current treatments for these disorders remain challenging. In fact, the current availability of diverse classes of mood stabilisers, antidepressants and antipsychotics to treat these disorders is plagued by many shortcomings, including delayed onset of action, intolerable side-effects and poor compliance. Around 40% of patients fail to respond to any of the typical frontline mood stabilisers or second-generation antipsychotics.^27,28^ Overall, the shortfalls in efficacy are attributable to the multifactorial nature of these conditions,^29^ and so new treatments should be similarly multimodal.^30^ Multimodal drugs, including vortioxetine^31^ and agomelatine,^32^ continue to gain favour, and many herbal substances have demonstrated antidepressant effects in preclinical and clinical studies, often with mechanisms similar to that of standard antidepressants.^33,34^ Additionally, these preparations may exhibit multitarget mechanisms of action on many different neurobiological processes implicated in mood disorders. For example, the multimodal actions predicted to underlie rapid and improved antidepressant response of drugs lsuch as ketamine include, among others, simultaneous modulation of 5-HT neuronal activity and glutamate/_γ_-amino butyric acid balance, and the activation of the brain-derived neurotrophic factor–mammalian target of rapamycin pathway,^35^ leading to improved neuroplasticity and cognition. These actions may have relevance for the primarily cognitive benefits of mangosteen extract observed in this study.

Elaborating on the study rationale, people experiencing depression with suicidal features show low cerebrospinal fluid (CSF) levels of the metabolite of 5-HT and increased 5-HT2A receptor binding in platelets and prefrontal cortical sites.^3^ Similarly, post-mortem depressed bipolar disorder subjects have significantly reduced levels of 5-HIAA in frontal and parietal cortex, while depressed bipolar disorder individuals present with decreased CSF 5-HIAA levels, with manic sufferers showing elevated levels.^3^ Post-mortem studies in people with schizophrenia, and in psychotic patients, have observed reduced frontal cortex 5-HT2A receptor density, while CSF, genetic and neuroimaging studies have demonstrated an increase in central 5-HT-ergic neurotransmission in schizophrenia.^3^

Many constituents of mangosteen have bioactive effects that target the above dysregulated pathways in bipolar depression, particularly monoaminergic (notably serotonin), mitochondrial, immune-inflammatory and redox pathways.^3^ Moreover, this action may augment the therapeutic response of co-administered antipsychotics. Alpha-mangostin is the most common xanthone present in mangosteen pericarp and, along with beta- and gamma-mangostin, these are purported to be selective serotonin type 2A (5-HT2A) antagonists.^36^ 5-HT2A receptor antagonists are clinically effective antipsychotics, albeit as combined serotonin-dopamine antagonists such as clozapine and olanzapine.^37^ The latter are recognised for their improved efficacy in depressed mood and negative symptom schizophrenia.^3^ Through disinhibition of serotonin release, 5-HT2A receptor antagonists are also effective antidepressants (e.g. mianserin, mirtazapine).^3^ Animal studies have also described the serotonergic actions of mangosteen pericarp extract.^8^ Of interest here is that most of the adverse events reported in this study, while not differing from placebo, included headache, nausea, diarrhoea, suicidal ideation and migraine, which are typical side-effects experienced with the serotonin reuptake inhibitor class of antidepressants. Thus, mangosteen pericarp bolsters serotonin, be it either directly or indirectly.

Importantly, many mangosteen xanthones exhibit effective blood–brain barrier permeability.^38^ However, unlike other xanthones, alpha-mangostin exhibits relatively poor penetration of the blood–brain barrier^39^ in comparison with the much more permeable, but much less prevalent, gartanin, garcinone C and gamma-mangostin.^40^ However, the fact that chronic mangosteen pericarp displays significant antidepressant- and pro-cognitive effects in animals attests to the neurocognitive, and hence brain-penetrant, capabilities of the extract.^8^ Given that the current clinical trial investigated the ability of mangosteen pericarp to bolster existing treatment, our recent work showing comparable antidepressant effects in a prenatal maternal inflammation model is noteworthy in that both mangosteen pericarp and alpha-mangostin significantly bolstered the antidepressant-like response to haloperidol,^9^ in congruence with the findings described in this trial. This is not unlike the improved overall antidepressant, pro-cognitive and antipsychotic affects achieved with atypical antipsychotics, where the potent and selective dopamine D2 receptor blockade of haloperidol combines with the antiserotonergic actions of mangosteen pericarp extract to induce a more complete therapeutic response.

### Adverse effects

Adverse effects were evident in the mangosteen-treated group, amounting to 141 total adverse events compared with 137 in the placebo-treated group, with no significant differences between groups. This prevalence is not unlike that seen with other herbal preparations with a similar mechanism of action,^41^ as well as versus typical serotonergic antidepressants such as selective serotonin reuptake inhibitors.^42,43^ Of interest, among the most frequently reported adverse events were headache, nausea, diarrhoea, suicidal ideation and migraine, which are characteristic of selective serotonin reuptake inhibitors.^44^ Indeed, effects on 5HT have been described for mangosteen in earlier animal studies.^8^

### Limitations

The trial noted several limitations. Symptoms declined in both mangosteen and placebo groups early in the trial. Participants were required to have a minimum of 4 weeks of medication stability prior to commencement, and so mood improvement due to non-investigational drugs should have been relatively limited. This suggests that at least some of the improvement observed throughout the trial may be attributable to the Hawthorne effect, where participants’ knowledge of the trial contributed to general improvements. For example, participants may have been more mindful of their medication adherence or more likely to associate positive events with the trial itself,^45^ and future research could include additional post-intervention follow-ups. A second limitation of the study is the unclear influence of unstable treatment-as-usual in both conditions and a high degree of polypharmacy. Indeed, fewer than one quarter of participants were regularly taking a single medication (split almost entirely between antidepressants and mood stabilisers) at the beginning of the trial, and a further quarter were taking four or more. Changes to medication were also not uncommon, with between 5 and 10% of the sample changing antidepressants, mood stabilisers or antipsychotics at each trial visit. Individual variation in drug metabolism should be considered, and future investigation into the psychotropic benefits of mangosteen may need to be guided by pharmacogenetic analysis.

Our findings suggest that adjunctive mangosteen pericarp extract treatment can be superior to placebo in alleviating depressive, anxious and functional symptoms associated with bipolar disorder, but not manic symptoms or quality of life. Our lack of substantial omnibus effects probably reflects additional mechanisms that are not yet fully understood, although our observations of a significant overall benefit that did not persist following treatment washout give some cause for optimism. In this instance, mangosteen pericarp extract, via its various antioxidant, anti-inflammatory and neurogenic constituents, may explain the adjunctive beneficial effects observed in this study. Research involving larger samples and longer follow-up periods is necessary to both corroborate our promising findings and extend our understanding of both the potential benefits and risks associated with mangosteen pericarp in the treatment of bipolar depression.

## Supporting information

Dean et al. supplementary material Dean et al. supplementary material

## Data Availability

Data will be available through the SHeBA platform at Barwon Health. Please contact the corresponding author to access data.

## Supplementary material

The supplementary material is available online at https://doi.org/10.1192/bjp.2025.108

## References

[1] Dean OM , Gliddon E , Van Rheenen TE , Giorlando F , Davidson SK , Kaur M , et al. An update on adjunctive treatment options for bipolar disorder. Bipolar Disord 2018; 20: 87–96. 29369487 10.1111/bdi.12601

[2] Vieta E , Berk M , Schulze TG , Carvalho AF , Suppes T , Calabrese JR , et al. Bipolar disorders. Nat Rev Dis Primers 2018; 18008. 29516993 10.1038/nrdp.2018.8

[3] Brand SJ , Moller M , Harvey BH. A review of biomarkers in mood and psychotic disorders: a dissection of clinical vs. preclinical correlates. Curr Neuropharmacol 2015; 13: 324–68. 26411964 10.2174/1570159X13666150307004545 PMC4812797

[4] van Rensburg DJ , Lindeque Z , Harvey BH , Steyn SF. Reviewing the mitochondrial dysfunction paradigm in rodent models as platforms for neuropsychiatric disease research. Mitochondrion 2022; 64: 82–102. 35307580 10.1016/j.mito.2022.03.002

[5] Sarris J , Marx W , Ashton MM , Ng CH , Galvao-Coelho N , Ayati Z , et al. Plant-based medicines (phytoceuticals) in the treatment of psychiatric disorders: a meta-review of meta-analyses of randomized controlled trials: Les médicaments à base de plantes (phytoceutiques) dans le traitement des troubles psychiatriques: une méta-revue des méta-analyses d’essais randomisés contrôlés. Can J Psychiatry 2021; 66: 849–62. 33596697 10.1177/0706743720979917 PMC8573706

[6] Ashton MM , Dean OM , Walker AJ , Bortolasci CC , Ng CH , Hopwood M , et al. The therapeutic potential of mangosteen pericarp as an adjunctive therapy for bipolar disorder and schizophrenia. Front Psychiatry 2019; 10: 115. 30918489 10.3389/fpsyt.2019.00115 PMC6424889

[7] Ashton MM , Berk M , Ng CH , Hopwood M , Dodd S , Turner A , et al. Efficacy of adjunctive Garcinia mangostana Linn (mangosteen) pericarp for bipolar depression: study protocol for a proof-of-concept trial. Braz J Psychiatry 2019; 41: 245–53. 30328970 10.1590/1516-4446-2018-0114 PMC6794139

[8] Oberholzer I , Möller M , Holland B , Dean OM , Berk M , Harvey BH. Garcinia mangostana Linn displays antidepressant-like and pro-cognitive effects in a genetic animal model of depression: a bio-behavioral study in the flinders sensitive line rat. Metab Brain Dis 2018; 33: 467–80. 29101602 10.1007/s11011-017-0144-8

[9] Lotter J , Möller M , Dean O , Berk M , Harvey BH. Studies on haloperidol and adjunctive α-mangostin or raw Garcinia mangostana Linn pericarp on bio-behavioral markers in an immune-inflammatory model of schizophrenia in male rats. Front Psychiatry 2020; 11: 121. 32296347 10.3389/fpsyt.2020.00121 PMC7136492

[10] Chan A-W , Tetzlaff JM , Altman DG , Laupacis A , Gøtzsche PC , Krleža-Jerić K, et al. SPIRIT statement: defining standard protocol items for clinical trials. Ann Intern Med 2013; 158: 200–7. 23295957 10.7326/0003-4819-158-3-201302050-00583 PMC5114123

[11] American Psychiatric Association. Diagnostic and Statistical Manual of Mental Disorders. American Psychiatric Association, 2013.

[12] APA. Diagnostic and Statistical Manual of Mental Disorders: DSM-5™, 5th ed. American Psychiatric Publishing, Inc., 2013.

[13] Montgomery SA , Åsberg M. A new depression scale designed to be sensitive to change. Br J Psychiatry 1979; 134: 382–9. 444788 10.1192/bjp.134.4.382

[14] Berk M , Dodd S , Dean OM , Kohlmann K , Berk L , Malhi GS. The validity and internal structure of the Bipolar Depression Rating Scale: data from a clinical trial of N-acetylcysteine as adjunctive therapy in bipolar disorder. Acta Neuropsychiatr 2010; 22: 237–42. 26952834 10.1111/j.1601-5215.2010.00472.x

[15] Max H. The assessment of anxiety states by rating. Br J Med Psychol 1959; 32: 50–5. 13638508 10.1111/j.2044-8341.1959.tb00467.x

[16] Young RC , Biggs JT , Ziegler VE , Meyer DA. A rating scale for mania: reliability, validity and sensitivity. Br J Psychiatry 1978; 133: 429–35. 728692 10.1192/bjp.133.5.429

[17] Spearing MK , Post RM , Leverich GS , Brandt D , Nolen W. Modification of the Clinical Global Impressions (CGI) scale for use in bipolar illness (BP): the CGI-BP. Psychiatry Res 1997; 73: 159–71. 9481807 10.1016/s0165-1781(97)00123-6

[18] Viktrup L , Hayes RP , Wang P , Shen W. Construct validation of patient global impression of severity (PGI-S) and improvement (PGI-I) questionnaires in the treatment of men with lower urinary tract symptoms secondary to benign prostatic hyperplasia. BMC Urol 2012; 12: 30. 23134716 10.1186/1471-2490-12-30 PMC3503561

[19] Morosini PL , Magliano L , Brambilla L , Ugolini S , Pioli R. Development, reliability and acceptability of a new version of the DSM-IV Social and Occupational Functioning Assessment Scale (SOFAS) to assess routine social funtioning. Acta Psychiatr Scand 2000; 101: 323-9. 10782554

[20] Keller MB. The longitudinal interval follow-up evaluation. Arch Gen Psychiatry 1987; 44: 5408. 10.1001/archpsyc.1987.01800180050009 3579500

[21] Stevanovic D. Quality of Life Enjoyment and Satisfaction Questionnaire - short form for quality of life assessments in clinical practice: a psychometric study. J Psychiatr Ment Health Nurs 2011; 18: 744–50. 21896118 10.1111/j.1365-2850.2011.01735.x

[22] Harris PA , Taylor R , Thielke R , Payne J , Gonzalez N , Conde JG. Research electronic data capture (REDCap)—A metadata-driven methodology and workflow process for providing translational research informatics support. J Biomed Inf 2009: 42: 377–81. 10.1016/j.jbi.2008.08.010 PMC2700030 18929686

[23] Laupu WK. The efficacy of Garcinia mangostana L. (mangosteen) pericarp as an adjunctive to second-generation antipsychotics for the treatment of schizophrenia: a double blind, randomised, placebo-controlled trial. 2014. DOI: researchonline.jcu.edu.au/40097/

[24] Berk M , Copolov DL , Dean O , Lu K , Jeavons S , Schapkaitz I , et al. N-acetyl cysteine for depressive symptoms in bipolar disorder—a double-blind randomized placebo-controlled trial. Biol Psychiatry 2008; 64: 468–75. 18534556 10.1016/j.biopsych.2008.04.022

[25] Dean OM , Kanchanatawan B , Ashton M , Mohebbi M , Ng CH , Maes M , et al. Adjunctive minocycline treatment for major depressive disorder: a proof of concept trial. Austr N Z J Psychiatry 2017; 51: 829–40. 10.1177/0004867417709357 28578592

[26] Moher D , Hopewell S , Schulz KF , Montori V , Gøtzsche PC , Devereaux PJ , et al. Explanation and elaboration: updated guidelines for reporting parallel group randomised trials. J Clin Epidemiol 2010; 63: e1–37. 20346624 10.1016/j.jclinepi.2010.03.004

[27] Sienaert P , Lambrichts L , Dols A , De Fruyt J. Evidence-based treatment strategies for treatment-resistant bipolar depression: a systematic review. Bipolar Disord 2013; 15: 61–9. 23190379 10.1111/bdi.12026

[28] Tournier M , Neumann A , Pambrun E , Weill A , Chaffiol J-P , Alla F , et al. Conventional mood stabilizers and/or second-generation antipsychotic drugs in bipolar disorders: a population-based comparison of risk of treatment failure. J Affect Disord 2019; 257: 412–20. 31306992 10.1016/j.jad.2019.07.054

[29] Fountoulakis KN , Yatham LN , Grunze H , Vieta E , Young AH , Blier P , et al. The CINP guidelines on the definition and evidence-based interventions for treatment-resistant bipolar disorder. Int J Neuropsychopharmacol 2020; 23: 230–56. 31802122 10.1093/ijnp/pyz064 PMC7177170

[30] Paul SM , Potter WZ. Finding new and better treatments for psychiatric disorders. Neuropsychopharmacology 2024; 49: 3–9. 37582978 10.1038/s41386-023-01690-5 PMC10700311

[31] Mukku SSR , Nadella RK. Vortioxetine-induced switch - new drug with emerging old problems. J Geriatr Ment Health 2021; 8: 58–9.

[32] McGowan NM , Kim DS , de Andres Crespo M , Bisdounis L , Kyle SD , Saunders KE. Hypnotic and melatonin/melatonin-receptor agonist treatment in bipolar disorder: a systematic review and meta-analysis. CNS Drugs 2022; 36: 345–63. 35305257 10.1007/s40263-022-00911-7

[33] Fajemiroye JO , da Silva DM , de Oliveira DR , Costa EA. Treatment of anxiety and depression: medicinal plants in retrospect. Fundament Clin Pharmacol 2016; 30: 198–215. 10.1111/fcp.12186 26851117

[34] Kenda M , Kočevar Glavač N , Nagy M , Sollner Dolenc M. Medicinal plants used for anxiety, depression, or stress treatment: an update. Molecules 2022; 27: 6021. 36144755 10.3390/molecules27186021 PMC9500625

[35] Li Y-F. A hypothesis of monoamine (5-HT)–glutamate/GABA long neural circuit: aiming for fast-onset antidepressant discovery. Pharmacol Therapeut 2020; 208: 107494. 10.1016/j.pharmthera.2020.107494 31991195

[36] Kalick LS , Khan HA , Maung E , Baez Y , Atkinson AN , Wallace CE , et al. Mangosteen for malignancy prevention and intervention: current evidence, molecular mechanisms, and future perspectives. Pharmacol Res 2023; 188: 106630. 36581166 10.1016/j.phrs.2022.106630

[37] Kantrowitz JT. Targeting serotonin 5-HT2A receptors to better treat schizophrenia: rationale and current approaches. CNS Drugs 2020; 34: 947–59. 32783116 10.1007/s40263-020-00752-2

[38] Wang S-N , Li Q , Jing M-H , Alba E , Yang X-H , Sabaté R et al. Natural xanthones from Garcinia mangostana with multifunctional activities for the therapy of Alzheimer’s disease. Neurochem Res 2016; 41: 1806–17. 27038926 10.1007/s11064-016-1896-y

[39] Chen Z-L , Huang M , Wang X-R , Fu J , Han M , Shen Y-Q , et al. Transferrin-modified liposome promotes α-mangostin to penetrate the blood-brain barrier. Nanomed Nanotechnol Biol Med 2016; 12: 421–30. 10.1016/j.nano.2015.10.021 26711963

[40] Chitchumroonchokchai C , Riedl KM , Suksumrarn S , Clinton SK , Kinghorn AD , Failla ML. Xanthones in mangosteen juice are absorbed and partially conjugated by healthy adults. J Nutr 2012; 142: 675–80. 22399525 10.3945/jn.111.156992 PMC3301988

[41] Beaubrun G , Gray GE. A review of herbal medicines for psychiatric disorders. Psychiatr Serv 2000; 51: 1130–4. 10970915 10.1176/appi.ps.51.9.1130

[42] Edinoff AN , Akuly HA , Hanna TA , Ochoa CO , Patti SJ , Ghaffar YA , et al. Selective serotonin reuptake inhibitors and adverse effects: a narrative review. Neurol Int 2021; 13: 387–401. 34449705 10.3390/neurolint13030038 PMC8395812

[43] Ferguson JM. SSRI antidepressant medications: adverse effects and tolerability. Prim Care Companion J Clin Psychiatry 2001; 3: 227. 10.4088/pcc.v03n0105 PMC181155 15014625

[44] Harvey BH. The neurobiology and pharmacology of depression: a comparative overview of serotonin selective antidepressants. S Afr Med J 1997; 87: 540–52. 9180828

[45] Sedgwick P , Greenwood N. Understanding the Hawthorne effect. BMJ 2015; 351: h4672. 26341898 10.1136/bmj.h4672

