# Supplementary material for: The efficacy of adjunctive Garcinia mangostana Linn. (mangosteen) pericarp extract for bipolar depression: 24-week randomised controlled trial
Source: Br J Psychiatry. 2025 Jul 10;227(6):854–63. doi: 10.1192/bjp.2025.108 (PMC12628125; doi:10.1192/bjp.2025.108)
Supplement: Dean et al. supplementary material [file S0007125025001084sup001.docx]

**Table S1**

|  |  | Placebo | Mangosteen | Total |
| --- | --- | --- | --- | --- |
| AE ORGAN CODE | AE SYMPTOM | Count | Count | Count |
| Blood and lymphatic system disorders (10005329) | | | | |
|  | Chronic Lymphocytic Leukaemia (CLL) (10009310) | 1 | 0 | 1 |
|  | Hyperglycaemia (10020635) | 1 | 1 | 2 |
|  | Swollen glands (10042720) | 1 | 0 | 1 |
| Cardiac disorders (10007541) | | | | |
|  | Chest pains (10008479) | 0 | 1 | 1 |
| Ear and labyrinth disorders (10013993) | | | | |
|  | Ear infection (earache) (10014011) | 0 | 1 | 1 |
|  | Earache (10014037) | 0 | 1 | 1 |
|  | Hearing impaired (10019245) | 1 | 0 | 1 |
|  | Otitis media (10033078) | 1 | 0 | 1 |
|  | Tinnitus (10043882) | 1 | 1 | 2 |
|  | Improved vertigo (10047340) | 1 | 2 | 3 |
| Endocrine disorders (10014698) | | | | |
|  | Hypothyroidism (10021114) | 2 | 1 | 3 |
|  | Menstruation irregular (10027336) | 1 | 0 | 1 |
|  | Thyroglossal cyst (10051320) | 1 | 0 | 1 |
|  | Thyroid mass (10058900) | 1 | 0 | 1 |
| Eye disorders (10015919) | | | | |
|  | Blurred vision (10005886) | 0 | 1 | 1 |
|  | Eye irritation (10015946) | 0 | 1 | 1 |
| Gastrointestinal disorders (10017947) | | | | |
|  | Abdominal discomfort (10000059) | 1 | 1 | 2 |
|  | Abdominal pain (10000081) | 2 | 0 | 2 |
|  | Bloating (10005265) | 1 | 1 | 2 |
|  | Blood in stool (10005603) | 2 | 0 | 2 |
|  | Change of bowel habit (10008399) | 1 | 0 | 1 |
|  | Colitis (10009887) | 0 | 1 | 1 |
|  | Constipation (10010774) | 0 | 6 | 6 |
|  | Diarrhoea (10012735) | 4 | 9 | 13 |
|  | Diverticulitis (10013538) | 1 | 1 | 2 |
|  | Food poisoning (10016952) | 1 | 0 | 1 |
|  | Gastritis (10017853) | 1 | 0 | 1 |
|  | Gastroenteritis (10017888) | 6 | 2 | 8 |
|  | IBS (10017947) | 0 | 1 | 1 |
|  | Gastrointestinal pain (10017999) | 2 | 0 | 2 |
|  | Heartburn (10019326) | 2 | 1 | 3 |
|  | Indigestion (10021706) | 2 | 0 | 2 |
|  | Irritable bowel syndrome (10023003) | 1 | 0 | 1 |
|  | Nausea (10028813) | 9 | 8 | 17 |
|  | Obstruction gastric (bowel) (10029957) | 0 | 1 | 1 |
|  | Parotid duct obstruction (saliva gland) (10034021) | 1 | 0 | 1 |
|  | Stomachache (10042076) | 0 | 3 | 3 |
|  | Vomiting (10047700) | 1 | 3 | 4 |
|  | Gastrointestinal upset (10060962) | 1 | 1 | 2 |
|  | Duodenogastric reflux (acid reflux) (10060865) | 0 | 1 | 1 |
| General disorders and administration site conditions (10018065) | | | | |
|  | Breast swelling (10006312) | 1 | 1 | 2 |
|  | Fatigue (10016256) | 1 | 0 | 1 |
|  | Fever (10016558) | 2 | 0 | 2 |
|  | Sedation (10039897) | 1 | 0 | 1 |
|  | Sweating (10042661) | 1 | 1 | 2 |
|  | Fatigue (10043890) | 5 | 0 | 5 |
|  | Neck swelling (10049568) | 1 | 0 | 1 |
|  | Decreased appetite (10061428) | 3 | 2 | 5 |
| Hepatobiliary disorders (10019805) | | | | |
|  | Gall stones (10017650) | 0 | 1 | 1 |
| Immune system disorders (10021428) | | | | |
|  | Asthma (10003553) | 1 | 0 | 1 |
|  | Hay fever (10019170) | 3 | 2 | 5 |
|  | Hives (10020197) | 1 | 0 | 1 |
| Infections and infestations (10021881) | | | | |
|  | Bacterial food poisoning (10004022) | 0 | 1 | 1 |
|  | Bacterial vaginosis (10004055) | 1 | 0 | 1 |
|  | Infection (10021789) | 1 | 1 | 2 |
|  | Pharyngitis (10034835) | 0 | 1 | 1 |
|  | Pneumonia (10035664) | 0 | 1 | 1 |
|  | Sinusitis (10040753) | 0 | 2 | 2 |
|  | Skin infection (10040872) | 0 | 1 | 1 |
|  | Streptococcal sore throat (10042186) | 1 | 0 | 1 |
|  | Throat infection (10043520) | 1 | 0 | 1 |
|  | Tonsillitis (10044008) | 0 | 1 | 1 |
|  | Upper respiratory tract infection (10046306) | 1 | 0 | 1 |
|  | Urinary tract infection (10046571) | 1 | 4 | 5 |
|  | Viral infection (10047461) | 2 | 1 | 3 |
|  | Rhinovirus (10061494) | 0 | 1 | 1 |
|  | Ross river fever (10066922) | 1 | 0 | 1 |
|  | Tooth abscess (10074016) | 0 | 1 | 1 |
|  | Cold sore mouth (10049319) | 2 | 0 | 2 |
|  | Ear, nose and throat infection (upper respiratory tract infection with earache) (10072908) | 1 | 0 | 1 |
| Injury, poisoning and procedural complications (10022117) | | | | |
|  | Ankle sprain (10002549) | 1 | 0 | 1 |
|  | Burn (hand) (10006634) | 0 | 1 | 1 |
|  | Drug withdrawal syndrome (prescribed Seroquel) (10013754) | 1 | 0 | 1 |
|  | Fibula fracture (10016667) | 0 | 1 | 1 |
|  | Head injury (10019196) | 0 | 1 | 1 |
|  | Heat exhaustion (10019333) | 0 | 1 | 1 |
|  | Spider bite (10041517) | 0 | 1 | 1 |
|  | Sunburn (10042496) | 1 | 0 | 1 |
|  | Lithium toxicity (10050501) | 0 | 1 | 1 |
|  | Orbital fracture (10063638) | 1 | 0 | 1 |
|  | Skin abrasion (10064990) | 1 | 0 | 1 |
| Investigations (10022891) | | | | |
|  | Chromium decreased (1008798) | 1 | 0 | 1 |
|  | Blood cholesterol increased (10005425) | 1 | 0 | 1 |
|  | Colonoscopy (10010007) | 2 | 1 | 3 |
|  | Ferritin deficiency (10016457) | 1 | 0 | 1 |
|  | High cholesterol (10020049) | 1 | 0 | 1 |
|  | Nonspecific abnormal findings on radiological and other examination of lung field (10029685) | 1 | 0 | 1 |
|  | Weight gain (10047896) | 2 | 0 | 2 |
|  | Weight loss (10047900) | 2 | 0 | 2 |
|  | White blood cell counts increased (10047943) | 1 | 0 | 1 |
|  | White blood cell counts high (increased leukocytes) (10051771) | 0 | 1 | 1 |
|  | High platelet count (10074399) | 1 | 0 | 1 |
|  | Blood lithium level increased (10063687) | 1 | 0 | 1 |
| Metabolism and nutrition disorders (10027433) | | | | |
|  | Gluten intolerance (10018460) | 1 | 0 | 1 |
|  | Hypercholesterolemia (10020604) | 1 | 0 | 1 |
|  | Hypoglycaemia (10020993) | 2 | 0 | 2 |
|  | Hypokalaemia (10021018) | 1 | 0 | 1 |
|  | Vitamin B12 deficiency (10047609) | 1 | 0 | 1 |
|  | Vitamin B deficiency (10047626) | 5 | 0 | 5 |
|  | Type II diabetes (10067585) | 0 | 1 | 1 |
| Musculoskeletal and connective tissue disorders (10028395) | | | | |
|  | Back pain (10003988) | 3 | 3 | 6 |
|  | Bruising (10006504) | 1 | 0 | 1 |
|  | Costochondritis (10011219) | 1 | 0 | 1 |
|  | Foot pain (10016974) | 1 | 0 | 1 |
|  | Groin pain (10018735) | 1 | 0 | 1 |
|  | Joint ache (10023197) | 1 | 0 | 1 |
|  | Joint pain (10023222) | 0 | 1 | 1 |
|  | Knee pain (10023477) | 1 | 1 | 2 |
|  | Leg cramps (10024125) | 1 | 0 | 1 |
|  | Leg pain (10024130) | 1 | 0 | 1 |
|  | Ligament strain (toe) (10024453) | 0 | 1 | 1 |
|  | Muscle cramps (10028295) | 0 | 1 | 1 |
|  | Muscle pain (10028322) | 2 | 0 | 2 |
|  | Muscle spasms (10028334) | 1 | 0 | 1 |
|  | Muscular skeletal pain (10028391) | 1 | 0 | 1 |
|  | Neck pain (10028836) | 0 | 1 | 1 |
|  | Pain in fingers (10033428) | 1 | 0 | 1 |
|  | Ligament injury (10061223) | 0 | 2 | 2 |
|  | Popliteal cyst rupture (10068078) | 0 | 1 | 1 |
|  | Shoulder pain (20000223) | 1 | 0 | 1 |
|  | Fibromyalgia (10048439) | 1 | 1 | 2 |
|  | Enthesitis (10058497) | 1 | 0 | 1 |
| Neoplasms benign, malignant and unspecified (incl cysts and polyps) (10029104) | | | | |
|  | Plantar myofibroma (10075377) | 0 | 1 | 1 |
| Nervous system disorders (10029205) |  |  |  |  |
|  | Agitation (10001497) | 1 | 0 | 1 |
|  | Aura (10003791) | 1 | 0 | 1 |
|  | Dizziness (10013573) | 2 | 2 | 4 |
|  | Extrapyramidal symptoms (10015835) | 1 | 0 | 1 |
|  | Headache (10019211) | 12 | 7 | 19 |
|  | Memory impairment (10027175) | 1 | 0 | 1 |
|  | Migraine (10027599) | 2 | 7 | 9 |
|  | Restless leg syndrome (10038741) | 0 | 1 | 1 |
|  | Restlessness (of leg) (10038743) | 0 | 1 | 1 |
|  | Sciatica (10039674) | 0 | 1 | 1 |
|  | Tingling feet/hands (10043877) | 1 | 0 | 1 |
|  | Tremor (10044577) | 0 | 1 | 1 |
|  | Trigeminal neuralgia (10044652) | 1 | 0 | 1 |
|  | Nerve injury (nerve pain in breast) (10052897) | 1 | 0 | 1 |
| Psychiatric disorders (10037175) | | | | |
|  | Amnesia (10001949) | 0 | 1 | 1 |
|  | Depression (10012378) | 4 | 3 | 7 |
|  | Elevated mood (10014483) | 1 | 1 | 2 |
|  | Hallucination (10019063) | 0 | 2 | 2 |
|  | Hypomania (10021030) | 2 | 5 | 7 |
|  | Insomnia (10022437) | 1 | 0 | 1 |
|  | Irritable (10023000) | 1 | 0 | 1 |
|  | Libido decreased (10024419) | 1 | 0 | 1 |
|  | Mania (10026749) | 0 | 3 | 3 |
|  | Manic episode (10026778) | 1 | 0 | 1 |
|  | Nightmares (10029414) | 1 | 0 | 1 |
|  | Panic attack (10033664) | 2 | 0 | 2 |
|  | Psychotic (10037248) | 2 | 0 | 2 |
|  | Sleep disturbance (10040995) | 1 | 0 | 1 |
|  | Stress (increased PTSD symptoms) (10042209) | 0 | 1 | 1 |
|  | Suicidal ideation (10042458) | 4 | 5 | 9 |
|  | Stress (10049901) | 1 | 0 | 1 |
|  | Lithium toxicity (10070863) | 0 | 1 | 1 |
|  | Distress (restrictive eating) (10049119) | 0 | 1 | 1 |
| Renal and urinary disorders (10038359) | | | | |
|  | Kidney disorder (10013231) | 1 | 0 | 1 |
|  | Dysuria (10013990) | 0 | 2 | 2 |
|  | Fluid retention (10016807) | 1 | 0 | 1 |
|  | Incontinence (10021639) | 1 | 0 | 1 |
|  | Elevated LFTs (10077692) | 0 | 2 | 2 |
| Respiratory, thoracic and mediastinal disorders (10038738) | | | | |
|  | Bronchitis (10006451) | 0 | 1 | 1 |
|  | Chest infection (10008477) | 4 | 6 | 10 |
|  | Cold (10009851) | 14 | 16 | 30 |
|  | Cough (10011224) | 1 | 1 | 2 |
|  | Difficulty breathing (lung tension) (10012791) | 1 | 0 | 1 |
|  | Flu (10016790) | 3 | 8 | 11 |
|  | Shortness of breath (10040604) | 0 | 1 | 1 |
|  | Sinus infection (10040745) | 2 | 0 | 2 |
|  | Sore throat (10041367) | 2 | 0 | 2 |
|  | Viral upper respiratory tract infections (10047483) | 0 | 1 | 1 |
|  | Bronchitis (10052613) | 3 | 1 | 4 |
|  | Chronic sore throat (10066666) | 0 | 1 | 1 |
|  | Dermatitis (10012431) | 0 | 1 | 1 |
|  | Itchy scalp (10023093) | 0 | 1 | 1 |
|  | Itchy skin (10023094) | 1 | 1 | 2 |
|  | Plantar wart (10035158) | 1 | 0 | 1 |
|  | Rash (10037844) | 0 | 2 | 2 |
|  | Skin lesion (10040882) | 1 | 0 | 1 |
| Surgical and medical procedures (10042613) | | | | |
|  | TMS (10051505) | 1 | 0 | 1 |
|  | Medication dose changed (hospitalisation monitoring Seroquel reduction) (10064926) | 0 | 1 | 1 |
| Vascular disorders (10047065) |  |  |  |  |
|  | Bleeding nose (10005124) | 0 | 1 | 1 |
|  | Syncope (10011954) | 0 | 1 | 1 |
|  | Hypertension (worsening) (10020772) | 0 | 1 | 1 |
|  | Tachycardia (10043071) | 1 | 0 | 1 |
|  | Hot flush (10060800) | 0 | 1 | 1 |

**Table S2**

| Medication | Treatment condition | Sum of Net Change in daily dose (mg) over trial | Mean change  (mg) | SD  (mg) | N |
| --- | --- | --- | --- | --- | --- |
| *Antidepressants* |  |  |  |  |  |
| Agomelatine | Placebo | 0 | 0 | 43.3 | 3 |
|  | Mangosteen | -50 | -25 | 0 | 2 |
| Amitriptyline | Placebo | 60 | 30 | 28.28 | 2 |
| Bupropion | Placebo | -268 | -134 | 234.76 | 2 |
| Clomipramine | Placebo | 25 | 25 |  | 1 |
| Desvenlafaxine | Placebo | 50 | 50 |  | 1 |
|  | Mangosteen | -250 | -125 | 35.36 | 2 |
| Fluoxetine | Placebo | 20 | 20 |  | 1 |
|  | Mangosteen | -130 | -43.33 | 55.08 | 3 |
| Fluvoxamine | Placebo | -200 | -200 |  | 1 |
| Imipramine | Placebo | -25 | -25 |  | 1 |
| Sertraline | Placebo | -350 | -87.5 | 103.08 | 4 |
|  | Mangosteen | 150 | 75 | 35.36 | 2 |
| Tranylcypomine | Placebo | -20 | -20 |  | 1 |
|  | Mangosteen | 20 | 20 |  | 1 |
| Venlafaxine | Placebo | 75 | 25 | 229.13 | 3 |
|  | Mangosteen | -75 | -18.75 | 187.5 | 4 |
| Duloxetine | Mangosteen | -180 | -90 | 42.43 | 2 |
| Escitalopram | Mangosteen | 10 | 10 |  | 1 |
| Paroxetine | Mangosteen | -10 | -10 |  | 1 |
| *Mood stabilisers* |  |  |  |  |  |
| Carbmazepine | Placebo | -700 | -700 |  | 1 |
|  | Mangosteen | 100 | 100 |  | 1 |
| Lamotrigine | Placebo | -400 | -33.33 | 191.39 | 12 |
|  | Mangosteen | -175 | -29.17 | 160.01 | 6 |
| Lithium | Placebo | 1025 | 73.21 | 725.53 | 14 |
|  | Mangosteen | -2025 | -155.77 | 659.16 | 13 |
| Sodium Valproate | Placebo | -1300 | -260 | 827.19 | 6 |
|  | Mangosteen | -2700 | -900 | 360.56 | 3 |
| *Antipsychotics* |  |  |  |  |  |
| Amisulpride | Placebo | -100 | -100 |  | 1 |
| Aripiprazole | Placebo | 15 | 5 | 8.66 | 3 |
|  | Mangosteen | -10 | -3.33 | 17.56 | 3 |
| Lurasidone | Placebo | -80 | -80 |  | 1 |
|  | Mangosteen | 120 | 60 | 28.28 | 2 |
| Olanzapine | Placebo | -30 | -30 |  | 1 |
|  | Mangosteen | -25 | -12.5 | 3.54 | 2 |
| Pericyazine | Placebo | 60 | 60 |  | 1 |
| Quetiapine | Placebo | -1231.5 | -102.63 | 173.55 | 12 |
|  | Mangosteen | -1090 | -68.13 | 220.28 | 16 |
| Asenapin | Mangosteen | -17.5 | -8.75 | 8.84 | 2 |
